# Alphachloralose intoxication: A retrospective study on epidemiology, clinical presentation, and management in an adult emergency department in Morocco

**DOI:** 10.1016/j.afjem.2025.100887

**Published:** 2025-06-25

**Authors:** El Mehdi Samali, Abdelghafour El Koundi, Amine Meskine, Hicham Balkhi, Mohammed Moussaoui

**Affiliations:** a Anesthesiology service of Military Teaching Hospital Mohammed V, Faculty of Medicine and Pharmacy of Rabat, Mohammed V University, Morocco; b Medical Intensive Care of Military Teaching Hospital Mohammed V, Faculty of Medicine and Pharmacy of Rabat, Mohammed V University, Morocco; c Cardiac Anesthesiology and intensive care service, Military Teaching Hospital Mohammed V, Faculty of Medicine and Pharmacy of Rabat, Mohammed V University, Morocco; d Anesthesiology and Intensive Care Department, Military Teaching Hospital Mohammed V, Faculty of Medicine and Pharmacy of Rabat, Mohammed V University, Morocco; e Emergency Department, CHU IBN ROCHD, Faculty of Medicine and Pharmacy of Casablanca, HASSAN II University, Morocco

**Keywords:** Alphachloralose, Emergency medicine, Poisoning, Toxicology

## Abstract

**Background:**

Alphachloralose, initially used as a hypnotic and anesthetic, is now restricted to rodenticides. Despite limited medical use, it remains accessible in North Africa, contributing to intentional poisonings. Poisoning primarily presents with neurological and respiratory symptoms, posing a significant public health concern. This study describes the epidemiological, clinical, and therapeutic characteristics of alphachloralose poisoning cases admitted to the adult emergency department of a university hospital in Casablanca, Morocco.

**Methods:**

A retrospective study analyzed emergency department records for alphachloralose poisoning cases from October 2022 to June 2023. Poisoning was confirmed based on clinical presentation, witness accounts, and/or biological toxicological evidence. Data on demographics, exposure circumstances, clinical features, interventions, and outcomes were extracted. Severity was evaluated using the Poisoning Severity Score (PSS), grading the severity into five levels from 0 (no symptoms) to 4 (fatal). Hospital stay duration and complications were also assessed.

**Results:**

Some 53 cases were included, with mean age of patients 27 years, and a male-to-female ratio of 0.83. Suicidal ingestion accounted for the vast majority of cases (98 %). Alphachloralose was exclusively ingested in powdered form, as no other formulations are available in Morocco. Neurological and respiratory disturbances were the most common clinical manifestations, with 28 % of cases classified as severe according to the PSS. Gastric lavage was performed in 52.8 % of cases, benzodiazepines were administered in 54 %, and 39.6 % of patients required intubation. The median time to admission to intensive care was 5 h. The average duration of hospitalization was 2.4 ± 1.2 days. Although one fatality occurred, 98 % of cases recovered fully without complications, emphasizing the importance of early and appropriate management.

**Conclusion:**

Alphachloralose poisoning is a significant toxicological concern in North Africa due to its availability and misuse. Severe symptoms are frequent, but early intervention leads to favorable outcomes. Public health measures focusing on regulation and education are essential.

## African Relevance

• **Public Health Concern**: Alphachloralose intoxication represents a significant toxicological issue in North Africa, particularly due to its widespread availability and affordability in local markets, contributing to intentional poisonings.
• **Regional Epidemiological Insights**: The study offers valuable data on the prevalence, clinical manifestations, and outcomes of alphachloralose intoxication, addressing a knowledge gap in toxicology specific to Morocco and other Maghreb countries.
• **Resource-Limited Settings**: Highlights the challenges of managing acute poisoning cases in regions with limited access to advanced toxicological interventions and emphasizes the need for tailored strategies.
• **Vulnerable Populations**: Demonstrates the disproportionate impact on younger individuals and women, aligning with regional suicide trends and emphasizing the importance of targeted mental health interventions.
• **Policy Implications**: Underlines the necessity of stricter regulation and public education campaigns to mitigate misuse and reduce the burden of such intoxications.

## Introduction

Alpha-chloralose (C₈H₁₁Cl₃O₆), a rodenticide synthesized by condensing glucose with chloral, was first developed in 1889. It is a centrally acting sedative-hypnotic and neurotoxic agent that potentiates GABAergic inhibition and suppresses excitatory neurotransmission, leading to profound CNS depression. It primarily enhances GABA-A receptor activity [1] while also inhibiting glutamatergic NMDA receptors, resulting in hypnosis, analgesia, and hypothermia. Initially used in human medicine as a hypnotic and later as a general anesthetic and sedative [2], its medical use was discontinued due to its paradoxical sedative and stimulant effects on the central nervous system, including spontaneous myoclonic movements. In humans, alpha-chloralose can cause deep sedation, respiratory depression, bradycardia, and hypothermia, with overdose potentially leading to coma or death. It interacts with other CNS depressants such as benzodiazepines, barbiturates, and alcohol, amplifying their effects. Severe complications include seizures, cardiovascular instability, and metabolic acidosis, necessitating intensive supportive care in cases of poisoning. Despite these risks, its low cost and unrestricted accessibility have contributed to its continued non-medical use.

Alphachloralose is primarily marketed as a rodenticide in powder, pellet, and liquid formulations, with the powdered form being the most widely available in Morocco. Typically marketed as a powder in 3- and 7-gram sachets with a 70 % concentration, alphachloralose ranked as the third leading cause of pesticide poisoning in 2022, accounting for 6.2 % of cases, following pyrethroids and organophosphates [3]. In Morocco, alphachloralose is widely employed as a rodenticide in agriculture and domestic settings. It is freely sold in grocery stores, hardware shops, veterinary pharmacies, and rural markets at affordable prices.

The primary route of exposure in reported human cases is oral ingestion, although occupational or accidental exposure via inhalation or dermal contact is possible, with significantly lower systemic absorption in these cases.

The toxic dose for adults is approximately 1 *g*, while for children it is around 20 mg/kg [4]. The lethal dose is estimated at 0.1 g/kg [5]. Clinical symptoms, which correlate directly with the ingested dose, usually appear within minutes to hours depending on factors such as dose, formulation, co-ingested substances, gastric contents, and individual variability. Alphachloralose has a large volume of distribution, accumulating in the kidneys, liver, and central nervous system. It is primarily eliminated through urine (90 %) in a conjugated form, with 45 % excreted within 24 h. The drug’s half-life is 4 to 5 h with no cumulative effect [6,7].

Poisoning with alphachloralose is infrequently reported, largely due to its low incidence in Western literature. This study presents an analysis of alphachloralose poisoning in the adult emergency department at a University Hospital in Casablanca, Morocco.

## Material and methods

This retrospective observational study was conducted in the adult Emergency Department (ED) of Ibn Rochd University Hospital, a tertiary care center located in Casablanca, Morocco. The ED manages approximately 70,000 patients annually, serving a predominantly urban population with a mix of rural referrals. It is a regional referral center for various emergencies, including toxicological cases, trauma, cardiovascular conditions, and infectious diseases.

The study period spanned nine months, from October 2022, to June 2023. The study was conducted in accordance with the Declaration of Helsinki and was approved by the Institutional Review Board of Ibn Rochd University Hospital. Due to the retrospective design, informed consent was waived. Patient confidentiality was maintained through anonymized data during collection and analysis.

Inclusion criteria encompassed patients admitted due to confirmed alphachloralose ingestion, which was established through a combination of clinical presentation consistent with known features of alphachloralose poisoning (e.g., central nervous system depression, myoclonus), corroborated by at least one of the following: (1) identification of alphachloralose packaging or residues at the scene, (2) a history provided by the patient or relatives indicating intentional or accidental ingestion, or (3) toxicological analysis when available. Cases lacking sufficient clinical or contextual evidence to confirm exposure were excluded

For each case, data were systematically extracted from medical records using a standardized data collection form. Demographic characteristics, including age and sex, were documented. The circumstances of exposure, such as mode (e.g., oral ingestion, cutaneous exposition, inhalation) and intent (e.g., suicidal or accidental), were thoroughly reviewed. Potential confounding factors, such as co-ingestion of other substances (e.g., alcohol, benzodiazepines, or other sedatives), were assessed. Pre-existing medical conditions were considered in the analysis. Clinical presentations were categorized into distinct symptom groups: neurological signs (e.g., convulsive seizures, coma, Glasgow Coma Scale [GCS] scores), nicotinic signs (e.g., focal or generalized myoclonus, myosis), muscarinic signs (e.g., bronchial congestion, hypersalivation), cardiovascular signs (e.g., cardiac arrest, sinus bradycardia, hypotension), digestive signs (e.g., abdominal pain, vomitting) and systemic signs such as hypothermia.

Therapeutic interventions were recorded in detail, including the administration of activated charcoal, benzodiazepine therapy, use of gastric lavage, forced diuresis, intubation, and mechanical ventilation. The severity of each case was classified using the Poison Severity Score (PSS), a standardized tool used to assess the clinical impact of poisoning. The PSS categorizes poisoning severity into five levels: 0 (no symptoms), 1 (mild symptoms), 2 (moderate symptoms), 3 (severe or life-threatening symptoms), and 4 (fatal). This standardized classification system allows for objective assessment and facilitates comparisons across different studies and clinical settings [8]. Additionally, the post-exposure delay—the time elapsed between ingestion and time to initiation of treatment—was noted.

Patient outcomes were comprehensively assessed, with specific attention to admission to the intensive care unit (ICU), discharge status and mortality rates. Data abstraction was independently verified by two researchers to ensure accuracy. Data analysis was performed using IBM SPSS Statistics for Windows, Version 23.0.

Descriptive statistics were employed to summarize the data. Continuous variables were presented as means with standard deviations, while categorical variables were summarized as frequencies and percentages.

## Results

Over the study period, a total of 53 cases of alphachloralose poisoning were identified. The cohort included 24 males (45.3 %) and 29 females (54.7 %), with a male-to-female ratio of 0.83. The mean age of the patients was 27 years, with a range of 15 to 56 years. Voluntary poisoning, primarily due to suicidal intent, accounted for 98 % of the cases. One patient had a medical past history of asthma, while another one had undergone surgery.

**Fig. 1:**
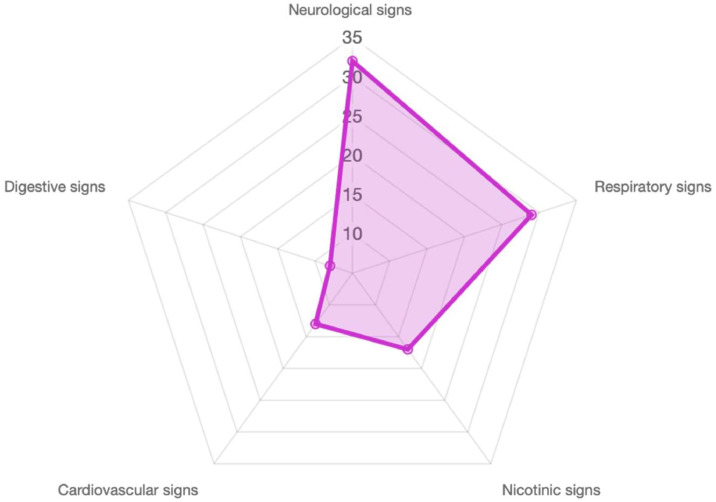
Distribution of clinical signs of alphachloralose poisoning (Values expressed in %).

Among the 53 cases, 84.9 % presented with clinical symptoms, and 28 % were classified as severe, based on the criteria of the PSS (Table 1). The patients exhibited predominantly neurological and/or respiratory disturbances (Fig. 1). The detailed manifestations are summarized in Table 2.

**Table 1:** Distribution of Cases by Poisoning Severity score (PSS).

PSS category	Total cases (n=53)	Percentage of total case (%)
PSS 0	15	28.3
PSS1	11	20.8
PSS 2	12	22.6
PSS 3	14	26.4
PSS 4	1	1.9

**Table 2:** Detailed clinical signs observed in patients with alphachloralose poisoning.

Clinical Sign	Number (%) of patients
**Neurological signs**	
Agitation	29 (54 %)
Convulsion	16 (30,2 %)
Coma	19 (35,8 %)
Headaches, diziness	21 (39,6 %)
**Respiratory signs**	
Respiratory depression	22 (41,5 %)
Bronchospasm	28 (52,8 %)
Hypersialorrhea	27 (50,9 %)
**Nicotinic signs**	
Mydriasis	20 (37,7 %)
Myoclonus, fasciculations	26 (49,1 %)
**Cardiovascular signs**	
Tachycardia	20 (37,7 %)
Bradycardia	5 (9,4 %)
Hypotension	12 (22,6 %)
**Digestive signs**	
Abdominal pain	11 (20,8 %)
Vomiting	11 (20,8 %)

**Fig. 2:**
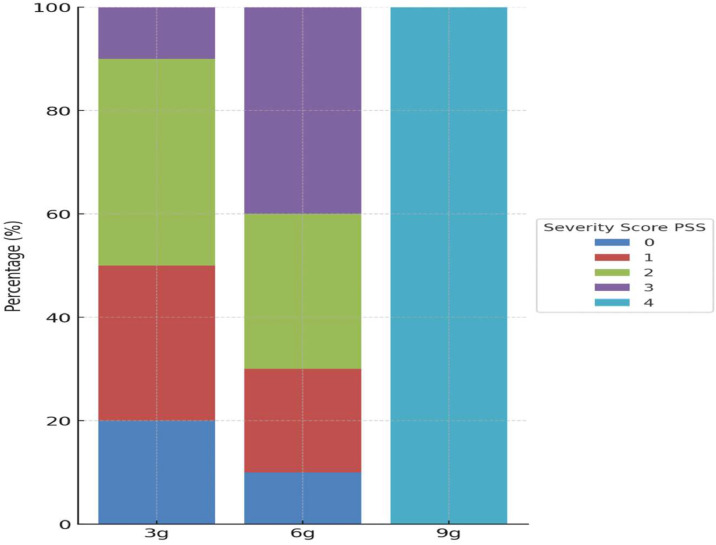
Distribution of the presumed ingested dose of Alphachloralose and corresponding Poisoning Severity Index Score.

A total of 38 patients ingested 3 *g* of alphachloralose, 14 ingested 6 *g*, and one patient ingested 9 *g* Fig. 2 illustrates the distribution of ingested doses of alphachloralose and their corresponding severity scores.

*Each bar represents the distribution of patients (in %) within a group*

The median time from ingestion to ICU admission was 5 h (mean ± SD: 5 ± 3.8 h; range: 1–24 h; interquartile range IQR: 2–8 h). Management strategies and referral decisions were tailored to the severity of the clinical presentation. Endotracheal intubation was required in 39.6 % of cases, benzodiazepines were administered in 54 %, gastric lavage was performed in 52.8 %, and activated charcoal was used in 22.6 %. The average duration of hospitalization was 2.4 ± 1.2 days (range: 1–7 days; IQR: 1.6–3.2 days). Ninety eight percent of cases recovered fully without severe complications. Notably, there was only one fatality, which involved a 34-year-old female who ingested 9 *g* of alpha-chloralose and experienced a delayed presentation to hospital (>10 h), requiring mechanical ventilation and vasopressor support.

## Discussion

Our study provides an in-depth analysis of alphachloralose poisoning within a North African context, emphasizing epidemiological trends, clinical manifestations, and therapeutic strategies. Voluntary alphachloralose poisoning in humans is rarely reported in the literature, primarily due to its low prevalence in Western countries, with an estimated annual incidence of approximately ten cases in France as reported by Richelme et al. In 1985 [9]. In contrast, the situation in North Africa is markedly different and aligns closely with our findings. In Tunisia, acute alphachloralose poisoning is reported much more frequently. According to Kouraichi et al., the intensive care unit of the Centre for Emergency Medical Assistance in Tunis—the main regional toxicology referral center—records an average of 100 hospitalizations for alphachloralose poisoning each year [6].

Handling alphachloralose-based rodenticides in occupational settings poses minimal risk, as absorption does not occur through the skin or respiratory tract [7]. Additionally, a predominance of female patients was observed, aligning with findings from other studies conducted in both Maghrebian and Western contexts [10,11]. The high rate of suicidal ingestion, particularly among young females, highlights a pressing need for strengthened mental health support, early identification of at-risk individuals, and targeted community awareness initiatives. These efforts are crucial to addressing the underlying psychosocial drivers of intentional poisoning. In parallel, establishing national surveillance systems and enforcing stricter regulation of over-the-counter access to rodenticides such as alphachloralose should be prioritized. These public health measures would help monitor poisoning trends and reduce the availability of high-risk substances in vulnerable communities.

Clinically, initial symptoms often include behavioral disturbances, a state resembling inebriation, and balance impairments. Depending on the ingested dose, these can progress to altered levels of consciousness, starting with a calm coma (Barbiturate-like), followed by a phase of motor agitation and neuromuscular hyperexcitability. This hyperexcitability is a hallmark of alphachloralose intoxication and is marked by myoclonus, which can escalate to generalized seizures triggered by minimal stimuli. In most cases, hypothermia, excessive bronchial secretions, and sinus tachycardia are also present, strongly suggesting chloralose poisoning. Other, less specific manifestations of central toxic effects include bradypnea, alveolar hypoventilation, respiratory pauses and pupillary abnormalities.

Neurological and respiratory symptoms were the most frequently observed clinical manifestations in our study, consistent with existing literature [[10], [11], [12], [13], [14]]. The PSS effectively stratified cases, with 28 % classified as severe aligning with existing research on dose-response relationships in alphachloralose poisoning [14]. The rapid absorption of chloralose, with symptoms typically appearing within 90 min [15], underscores the importance of early recognition and intervention. Myoclonus, seizures, and coma were key indicators of severe poisoning, reinforcing the need for vigilant monitoring in suspected cases.

The clinical course is usually rapidly favorable, with the prognosis largely depending on the promptness of medical intervention. Coma is typically short, lasting between 8 and 12 h, and recovery is generally complete without long-term sequelae. While 15 cases were associated with the ingestion of 6 *g* or more - considered the lethal dose for a 60 kg individual - our study and others [10,11,16] report low mortality rates. This may likely results from significant improvements in awareness, timely medical intervention and reversible toxic effects. Clinicians should focus on early recognition of symptoms, prompt supportive care, and research to refine management protocols.

Supportive care remains the cornerstone of management. The absence of a specific antidote underscores the importance of symptomatic management. The main objective of symptomatic treatment is to maintain the integrity of vital functions and to stop myoclonus or convulsions. Intubation and mechanical ventilation are required in cases of impaired consciousness and/or respiratory depression. When initiated early, they provide protection of the upper airways and reduce the risk of aspiration. Agitation, myoclonic manifestations, or seizures must be effectively controlled and benzodiazepines are commonly used for this purpose.

The high use of gastric lavage in our institution requires further discussion. Its continued use in our setting may reflect local clinical practices, and physician discretion in severe poisoning cases. Current international guidelines on pesticide poisoning, such as those from the World Health Organization (WHO) and the European Association of Poisons Centres and Clinical Toxicologists (EAPCCT), recommend early supportive care and generally discourage the use of gastric lavage unless it is performed within one hour of ingestion, and only if the patient is at significant risk of serious toxicity [17,18]. However, no well designed randomized prospective studies have been conducted to validate or refute the effectiveness of gastric lavage in pesticide poisonning [19] and Chloralose poisoning particularly. Until more evidence is available, gastric lavage should NOT be routinely used in pesticide exposure management as there is further evidence that lavage may propel the material into the small bowel, thus increasing absorption [20].

Administration of activated charcoal is generally recommended as a single dose (50 g) within the first two hours after ingestion and in the absence of contraindications [21].

Although most patients recovered without sequelae, long-term follow-up was not systematically conducted. In previous studies, alphachloralose poisoning has been associated with transient neurological impairments, but data on persistent effects remain scarce [16]. Future research should explore long-term outcomes to better understand the full spectrum of recovery and potential delayed complications.

This study has several limitations. Its retrospective design introduces the potential for information bias. Biological confirmation of poisoning was not systematically performed, and diagnosis sometimes relied on clinical judgment or contextual clues or witness accounts. Additionally, being a single-center study, the findings may not be generalizable to other settings.

## Conclusion

Alphachloralose poisoning represents a significant toxicological concern in our region, particularly due to its widespread availability and misuse in rural and underserved communities. Raising public awareness and implementing stricter regulations on the sale of alphachloralose are critical steps to prevent intentional and accidental poisonings.

Enhancing diagnostic capacity through clinician education, standardized treatment protocols, and broader access to toxicological screening tools can support early recognition and timely intervention. This would improve patient outcomes and reduce the likelihood of severe complications.

Finally, multicenter studies and long-term follow-up are needed to deepen our understanding of the clinical trajectory and potential sequelae of alphachloralose poisoning.

## CRediT authorship contribution statement

**Mohammed Moussaoui:** Conceptualization, study design, data Collection. **El Mehdi Samali:** Data collection, clinical insights, and manuscript drafting. **Abdelghafour El Koundi:** Data collection, clinical insights, and manuscript drafting. **Amine Meskine and Hicham Balkhi:** Statistical analysis, interpretation of findings, and manuscript editing.

All authors have reviewed and approved the final version.

## Funding

This research received no specific grant from any funding agency in the public, commercial, or not-for-profit sectors.

## Dissemination of findings

The findings of this research were shared through: presentations at local medical and toxicology conferences to inform healthcare professionals, engagement with public health authorities in Morocco to advocate for stricter regulations on the sale of alphachloralose, and through community outreach programs in rural and urban areas to raise awareness about the dangers of alphachloralose misuse and to promote mental health resources.

## Declaration of competing interest

The authors declare that they have no known competing financial interests or personal relationships that could have appeared to influence the work reported in this paper.
